# Partial-year continuous light treatment reduces precocious maturation in age 1+ hatchery–reared male spring Chinook Salmon (O*ncorhynchus tshawytscha*)

**DOI:** 10.1093/conphys/coac085

**Published:** 2023-01-20

**Authors:** Nick F Hoffman, Lea R Medeiros, Neil D Graham, Hayley M Nuetzel, Andrew L Pierce, James J Nagler

**Affiliations:** Department of Biological Sciences, University of Idaho, 875 Perimeter Dr., Moscow, ID 83844, USA; Department of Biological Sciences, University of Idaho, 875 Perimeter Dr., Moscow, ID 83844, USA; Columbia River Inter-Tribal Fish Commission, Fishery Science Department, 700 NE Multnomah St., Suite 1200, Portland, OR 97232, USA; Columbia River Inter-Tribal Fish Commission, Fishery Science Department, 700 NE Multnomah St., Suite 1200, Portland, OR 97232, USA; Department of Biological Sciences, University of Idaho, 875 Perimeter Dr., Moscow, ID 83844, USA; Columbia River Inter-Tribal Fish Commission, Fishery Science Department, 700 NE Multnomah St., Suite 1200, Portland, OR 97232, USA; Department of Biological Sciences, University of Idaho, 875 Perimeter Dr., Moscow, ID 83844, USA

**Keywords:** sex, photoperiod, hatchery

## Abstract

Hatchery programs designed to conserve and increase the abundance of natural populations of spring Chinook Salmon O*ncorhynchus tshawytscha* have reported high proportions of males precociously maturing at age 2, called minijacks. High proportions of minijacks detract from hatchery supplementation, conservation and production goals. This study tested the effects of rearing juvenile Chinook Salmon under continuous light (LL) on minijack maturation in two trials. The controls were maintained on a simulated natural photoperiod for both trials. For trial 1, LL treatment began on the summer solstice 2019 or the autumn equinox 2019 and ended in late March 2020 (LL-Jun-Apr and LL-Sep-Apr, respectively). A significant reduction in the mean percent of minijacks (%MJ) was observed versus control (28.8%MJ) in both LL-Jun-Apr (5.4%MJ) and LL-Sep-Apr (9.3%MJ). Trial 2 was designed to evaluate whether stopping LL treatment sooner was still effective at reducing maturation proportions relative to controls. LL treatments began on the summer solstice 2020 and continued until the winter solstice (LL-Jun-Dec) or the final sampling in April 2021 (LL-June-Apr). LL-Jun-Dec tanks were returned to a simulated natural photoperiod after the winter solstice. Both photoperiod treatments showed a significant reduction in mean %MJ from the control (66%MJ): LL-Jun-Dec (11.6%MJ), LL-Jun-Apr (10.3%MJ). In both trials, minijacks had higher body weights, were longer and had increased condition factor when compared to females and immature males in all treatment groups at the final sampling. In both trials, there was little or no effect of LL treatment on fork length or body weight in immature males and females versus controls, but an increase in condition factor versus controls was observed. This study shows that continuous light treatment reduces minijack maturation in juvenile male spring Chinook Salmon and could provide an effective method for Spring Chinook Salmon hatcheries interested in reducing minijack production.

## Teaser

Conservation hatcheries aim to increase the abundance of imperiled populations of spring Chinook Salmon (Oncorhynchus tshawytscha). However, these hatcheries produce large numbers of precociously maturing males, which detracts from hatchery production and conservation goals. This study shows that photoperiod manipulation can strongly reduce the number of precocious males.

## Introduction

Chinook Salmon *Oncorhynchus tshawytscha* express a diversity of life history pathways, which not only facilitates species resiliency but also results in fitness trade-offs (Bourret *et al.*, 2016). For example, in Chinook Salmon (and many other species), age at first maturity is phenotypically plastic and influenced by a range of abiotic and biotic factors (Healey, 1991; Taranger *et al.*, 2010). While variation in age at first maturity increases population resilience against disastrous environmental events in a particular year (Greene *et al.*, 2010; Satterthwaite *et al.*, 2017), earlier maturing individuals often experience reduced reproductive success (Berejikian *et al.*, 2010; Ford *et al.*, 2012). In anadromous Chinook Salmon, adults typically mature at age 4; however, a notable portion of the males mature at age 3 (jack; Healey, 1991). In addition to the jack life history form, precocious maturation in freshwater can occur in Chinook Salmon males at age 1 (microjacks) and age 2 (minijacks, Larsen *et al.*, 2013). It was previously believed that smoltification and precocious maturation were mutually exclusive in Chinook salmon (Foote *et al.*, 1991). However, there is evidence that precociously maturing males can show signs of smolt-like behaviour but are physiologically different from non-mature smolts (Larsen *et al.*, 2010). In contrast to males, precocious maturation at age 1 or 2 is extremely rare in Chinook Salmon females within their native range (Healey, 1991). In naturally spawning Chinook Salmon populations, microjacks and minijacks use a “sneaker” tactic to gain access to much larger anadromous females and successfully fertilize a portion of spawned ova (Ford *et al.*, 2015). This life history strategy reduces the mortality associated with leaving freshwater and living multiple years in the ocean and is maintained, albeit at a low level, within many populations by frequency dependent selection (Berejikian *et al.*, 2010; Schroder *et al.*, 2012).

In salmonid hatchery programs, however, elevated levels of precocious male maturation compared to wild populations are often observed (Larsen *et al.*, 2004). This is thought to be due to the accelerated growth rate and high energy reserves resulting from increased food availability in the hatchery environment (Larsen *et al.*, 2006; Taranger *et al.*, 2010). In the Columbia River Basin, hatchery programmes motivated by conservation goals have been established to increase the abundance of spring Chinook Salmon stocks listed under the US Endangered Species Act. These conservation hatcheries use a broodstock management strategy designed to minimize genetic divergence from the naturally spawning population they are meant to enhance (Mobrand *et al.*, 2005; Paquet *et al.*, 2011; Fast *et al.*, 2015; Waters *et al.*, 2015). However, these hatcheries often produce more minijacks (up to 70% of the male juveniles produced annually; Harstad *et al.*, 2014) than what is observed in the wild (Larsen *et al.*, 2010). The high proportion of minijacks from hatcheries reduces the total number of anadromous males available for the fishery that could be produced. Therefore, methods to reduce the high proportion of minijacks in these hatchery programs are needed.

Previous studies have often focused on feed manipulation or ration reduction as a mechanism to reduce precocious maturation rates (Larsen *et al.*, 2006; Galbreath *et al.*, 2020; Pierce *et al.*, 2021). While reduced feed ration during summer and fall months was effective in reducing minijack maturation, it also resulted in an undesirable reduction in the size of immature fish produced (Larsen *et al.*, 2006), necessitating the investigation of treatments that would not negatively impact growth. Photoperiod is a key environmental regulator of maturation in salmonids and modifying circannual rhythms through photoperiod manipulation is highly effective at controlling the timing of puberty in salmonids (Bromage *et al.*, 1984; Duston and Bromage, 1988). Continuous light treatment has been used in a variety of salmonid species to control unwanted early sexual maturation (Taranger *et al.*, 2010). Furthermore, several studies in different species of salmonids have demonstrated that, in the hatchery setting, continuous light treatments can effectively reduce the rate of male precocious maturation in fall spawning species. In Arctic charr (*Salvelinus alpinus*), continuous light beginning and ending at different times of the year resulted in a range of male precocious maturation proportions, with the best outcome resulting from an autumn start date and a return to a natural photoperiod in the spring (Liu and Duston, 2016, 2018). Overwinter continuous light treatments in Atlantic salmon (*Salmo salar*) also resulted in a decrease in the number of fish precociously maturing (Taranger *et al.*, 1999; Schulz *et al.*, 2006; Leclercq *et al.*, 2010). Previous work in Chinook Salmon showed that continuous light treatments beginning in the fall and continuing for nearly a full year can decrease the proportions of both female and male fish precociously maturing as 2-year-olds (Unwin *et al.*, 2005).

The aim of this study was to test whether partial-year continuous light treatments can reduce the proportion of male spring Chinook Salmon precociously maturing as minijacks assuming the constraints associated with a conservation hatchery setting. Two trials, in successive years, tested continuous light treatments of 6 to 10 months with different start times (summer solstice and fall equinox) and end times (winter solstice and near the spring equinox). The proportion of resulting minijacks was based on the measurement of elevated plasma levels of the male sex hormone 11-ketotestosterone at the termination of the trials (Medeiros *et al.*, 2018).

## Materials and Methods

### Study fish

The protocol for sampling design and fish care for this study was in accordance with and approved by the University of Idaho Animal Care and Use Committee. Adult spring Chinook Salmon were collected at the Roza Adult Monitoring Facility, WA (river kilometre 206 on Yakima River) from April to September (2018 and 2019) and transported to the Cle Elem Supplementation Research Facility (CESRF, river kilometre 297 on Yakima River; Cle Elum, WA.). These fish were progeny from crosses of first-generation hatchery origin anadromous parents (i.e. SH line; Fast *et al.*, 2015, Waters *et al.*, 2015). Gametes were obtained from these adults in September and used to produce progeny for this study in 2018 (trial 1, *n* = 2880) and 2019 (trial 2, *n* = 2400). Juveniles, 6 months post spawned (averaging 255.5 mg in body weight and 32.6 mm in fork length) were transported to the University of Idaho Aquaculture Research Institute (ARI; Moscow, ID) in March of each year. Upon arrival at the ARI, fish were randomly split between two 340-L troughs (dimensions = 3.5-m long × 0.6-m wide × 0.3-m depth) and fed to satiation every 1–2 hours during working hours every day. The troughs were maintained at 14–15°C by an inline chiller on a recirculating water system. The fish were maintained on a simulated natural photoperiod with an abrupt light and dark switch prior to the distribution into experimental tanks.

### Experimental design

Trial 1 was conducted from 20 June 2019 to 26 March 2020, and trial 2 was conducted from 21 June 2020 to 2 April 2021. The end dates for the trials were selected because by this time maturation status can accurately be assessed for fish spawning in the fall (Medeiros *et al.*, 2018). For both trials, 24 light-proof tanks (60 litres each) on a dedicated recirculating water system were maintained at 14.5–15°C by an inline chiller on a recirculating system. Through both trials, alkalinity ranged from 250 to 110 ppm, mean pH was 7.95 and hardness ranged from 228 to 165 mg CaCO_3_/L. Each tank was covered with a light proof lid. Artificial lighting was supplied by A160WE Tuna Sun aquarium lights (Kessil Aquarium; Richmond, CA) mounted on the underside of each lid. Photoperiod duration and intensity (1000 lux at water surface and 300 lux at tank bottom) were controlled by A-Series Spectral controllers (Kessil Aquarium, Richmond, CA) set up in series. Fish were fed Bio-Oregon pellets (www.bio-oregon.com) to satiation twice daily from Monday to Friday and once daily on Saturday and Sunday. Pellet size was adjusted appropriately for fish size throughout the trials.

**Figure 1:**
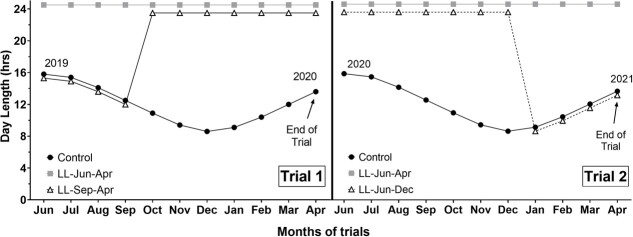
Photoperiod regimes for trial 1 (left panel) and trial 2 (right panel). Control is a simulated natural photoperiod and treatment lines indicate the period of time during which spring Chinook Salmon (*Oncorhynchus tshawytscha*) were exposed to continuous 24-hour light for each photoperiod treatment. The photoperiod treatment lines are depicted as offset by ±0.5 hours for visual clarity only; actual day length was as described in the Materials and Methods.

For trial 1, in June 2019, parr with a mean fork length [FL] = 72.0 ± 0.7 mm standard error (SE) and mean body weight [BW] = 3.89 ± 0.1 g SE) were randomly distributed into the experimental tanks (*n* = 120 per tank) and assigned to one of three photoperiod regimes (8 replicate tanks for each treatment): a simulated natural photoperiod (control, 46.7324°N, 117.0002°W), continuous (24-hour) light maintained from 21 June 2019 to 23 March 2020 (LL-Jun-Apr), and a simulated natural photoperiod from 21 June 2019 through 23 September 2019 then switched to continuous light until 23 March 2020 (LL-Sep-Apr; Figure 1, left panel). The final sampling was planned for early April but was shifted earlier due to the COVID-19 pandemic.

For trial 2, in June 2020, parr (FL = 75. ± 0.8 mm SE, BW = 5.1 ± 0.2 g SE) were randomly distributed into the same experimental tanks (*n* = 100 per tank) and subjected to the same rearing conditions as trial 1. The tanks were assigned one of three photoperiod regimes (eight replicate tanks for each treatment): a simulated natural photoperiod (control, 46.7324 N, 117.0002°W), continuous light maintained from 21 June 2020 to 2 April 2021 (LL-June-Apr), and continuous light from 21 June 2020 to 21 December 2020 then returned to simulated natural photoperiod until 2 April 2021 (LL-Jun-Dec; Figure 1, right panel).

### Sampling procedures

In trial 1, prior to sampling, fish were euthanized with an overdose of buffered tricaine methane sulfonate (MS-222; 0.1 g/L; Western Chemical, Ferndale, WA). Collection of FL (mm) and BW (g) for a subset of fish occurred on 23 September 2019 (*n* = 16 per tank) and 17 December 2019 (*n* = 20 per tank). Size parameters for the remaining fish were collected at the final sampling on 23–26 March 2020. Fulton’s condition factor (K) was calculated as: $K=100\times \mathrm{weight}\ \left(\mathrm{g}\right)\div \mathrm{fork}\ \mathrm{length}\ {\left(\mathrm{cm}\right)}^3$. At the final sampling, blood was taken by severing the caudal peduncle and collecting it from the dorsal vessel with a heparinized Natelson tube (Kimble Chase; Rockwood, TN). Blood was centrifuged at 7300 × *g* for 5 minutes and plasma was aspirated and stored at −80°C. Male fish were identified through dissection and visual inspection of gonads (control = 320, LL-June-Apr = 352, LL-Sept-Apr = 321, see Supplementary Table 1). To assess maturation status of male fish, 11-ketotestosterone (11-KT) was extracted from thawed plasma samples using ether extraction as previously described (Caldwell *et al.*, 2014). Reconstituted 11-KT samples were assayed in triplicate using an 11-KT Enzyme Linked Immunosorbent Assay (ELISA) Kit (Cayman Chemical; Ann Arbor, MI). Samples were diluted and re-assayed based on initial concentrations until values fell on the standard curve (20–80% binding). Intra/inter-assay coefficients of variation (%) for trial 1 and trial 2 were 6.27/6.67 and 3.67/9.78, respectively.

The fish in trial 2 were sampled as described above for trial 1. BW and FL were collected from a subset of individual fish on 21 December 2020 (*n* = 384, 16 per tank). Blood from males (*n*, control = 338, LL-June-Apr = 329, LL-Dec-Apr = 343) was sampled from the remaining fish during the final sampling period from 29 March to 2 April (see Supplementary Table 1).

### Data analyses

A modification of the method previously described by Medeiros *et al.* (2018) was used for assigning individual males into maturation categories based on plasma 11-KT values from samples collected at the final samplings for both trials. Analyses were performed in RStudio version 1.4.1106 (www.R-project.org). Modality of the 11-KT distributions was assessed using the excess mass test (Multimode package; https://arxiv.org/abs/1803.00472/). For distributions that were significantly bimodal, NormalMixEM (mixtools package; www.jstatsoft.org/v32/i06/), was used to determine a cutoff value between the modes. Those fish below the cutoff value were assigned as immature males (IMs) and those above as minijacks (MJs). All females were assigned as immature females (IFs). The significant bimodal distributions and cutoff values from the control groups were used to assign all of the males’ maturation status for both trials.

The proportion of males maturing as MJs for each treatment was calculated as $\mathrm{Proportion}\ \mathrm{MJ}=\big(\#\mathrm{MJ}\ \mathrm{males}$$\div \mathrm{total}\#\mathrm{males}\ \mathrm{per}\ \mathrm{tank}\big)$. Proportion data were arcsine-square root transformed prior to analysis and are presented as percentages. For other variables measured at the final sampling, after verifying that assumptions of normality were met prior to pooling individuals and calculating the means from each tank, individual values within a photoperiod treatment and maturation status category were averaged for each tank and used as inputs to the statistical analysis. BW and FL values were log_10_ transformed prior to analysis to meet the assumptions of normality. Thus, individual tanks from each treatment (*n* = 8 in each treatment) were used as the experimental units for these analyses.

Comparisons from the intermediate samplings for fish size and %MJ at final sampling between treatments were done with ordinary one-way analysis of variance (ANOVA) followed by Tukey’s multiple comparison test. Two-way ANOVA was used to test for effects of maturation category and treatment on BW, FL and K at the final samplings. Where significant interactions were found, ordinary one-way ANOVA was used to identify the effects of maturation category and treatment followed by Tukey’s multiple comparison test. A significance level of α = 0.05 was used for all ANOVA and multiple comparison analyses. All morphometric measurements are presented as mean ± SE. ANOVA analyses were completed in PRISM software version 9.1.1 (GraphPad Inc., La Jolla, CA).

## Results

**Figure 2:**
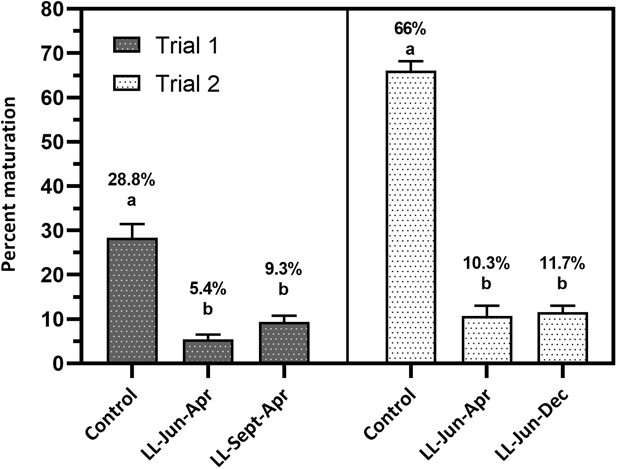
Comparison of %MJ spring Chinook Salmon (*Oncorhynchus tshawytscha*, proportion minijacks presented as percentages) across treatments for each trial. Tanks from each treatment (*n* = 8 in each treatment) used as experimental units. Mean and SE %MJ values. Different lowercase letters between treatments, within each trial, indicate significant differences (one-way ANOVA followed by Tukey’s multiple comparison test, *P* < 0.0001).

In both trials there were distinct bimodal distributions of plasma 11-KT in the control groups with little or no overlap between modes, which allowed 11-KT cutoff concentrations to be determined for each trial (trial 1 = 1.79 ng/mL, trial 2 = 2.56 ng/mL; see Supplementary figures 1-2 respectively). The distribution of plasma 11-KT concentrations for the continuous light treatments was not significantly bimodal (see Supplementary figures 3-6). Continuous light photoperiod treatments had a strong effect on reducing the %MJ at the end of both trials (One-way ANOVA, *P* < 0.0001; Figure 2). For both trials, the mean %MJ in the control group was significantly higher than the respective continuous light treatment groups. No significant differences in mean %MJ were detected between the continuous light treatments in either trial. Between years, the trial 2 control group had a higher mean %MJ (66%) compared to the trial 1 control group (mean = 28.8%). Of the 24-hour photoperiod regimes, trial 1 LL-Jun-Apr had the lowest mean %MJ (5.4%) and trial 2 LL-Jun-Dec the highest (mean = 11.7%). In trial 1, mean %MJs for LL-Sep-Apr was 9.3% and 10.3% for LL-Jun-Apr in trial 2.

**Figure 3:**
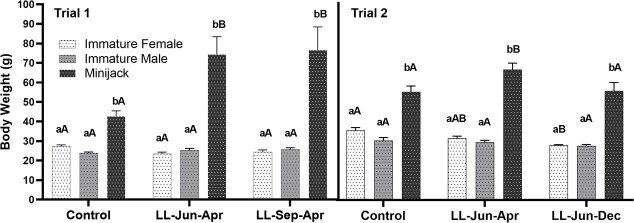
Comparisons of spring Chinook Salmon (*Oncorhynchus tshawytscha*) BW (mean ± SE) between treatment and maturation category for trial 1 (left panel) and trial 2 (right panel) in juvenile spring Chinook Salmon measured at final samplings. Lowercase letters indicate comparisons between maturation categories within a photoperiod treatment group (bars not sharing a letter differ significantly; one-way ANOVA followed by Tukey’s multiple comparison tests, *P* < 0.05). Uppercase letters indicate comparisons between photoperiod treatment groups within each maturation category (significance indication and statistical analysis same as for lowercase letters).

Two-way ANOVA revealed that the following factors had a strong effect on BW in trial 1 and trial 2: treatment, maturation category and the interaction between them (Table 1). The MJs, regardless of treatment, were significantly heavier than IFs and IMs at the end of both trials (Figure 3). MJs in the control groups weighed less (trial 1 = 42.7 g, trial 2 = 55.0 g) than MJs in both trials’ continuous light treatments, except in the trial 2 LL-Jun-Dec treatment (55.5 g). No significant differences in BW were detected between IMs and IFs within any of the photoperiod treatments. No significant differences in BW were detected between photoperiod treatments in IMs or IFs in trial 1. However, a significant reduction in BW was observed in IFs in trial 2’s LL-Jun-Dec (27.8 g) treatment versus the control treatment (35.5 g; one-way ANOVA followed by Tukey’s multiple comparison test, *P* = 0.0484).

**Table 1:** Results from two-way ANOVAs on the effects of photoperiod treatment, maturation category and the interaction of photoperiod treatment and maturation category on body weight (BW; g), fork length (FL; mm) and Fulton’s condition factor (K) in age 1+ male spring Chinook Salmon (*Oncorhynchus tshawytscha)* at the end of each trial.

**Response**	**Effect**	**SS**	**DF**	**F**	***P* value**
Trial 1 BW	Photoperiod treatment	1745	2	4.18	**0.0197**
	Maturation category	25 036	2	59.97	**<0.0001**
	Interaction	4076	4	4.881	**0.0017**
Trial 2 BW	Photoperiod treatment	357.5	2	4.389	**0.0164**
	Maturation category	13 211	2	162.2	**<0.0001**
	Interaction	579.4	4	3.557	**0.0112**
Trial 1 FL	Photoperiod treatment	429.5	2	1.856	0.1647
	Maturation category	22 675	2	98.02	**<0.0001**
	Interaction	2761	4	5.966	**0.0004**
Trial 2 FL	Photoperiod treatment	198.1	2	2.504	0.0899
	Maturation category	17 809	2	225.1	**<0.0001**
	Interaction	1225	4	7.739	**<0.0001**
Trial 1 K	Photoperiod treatment	0.139	2	29.82	**<0.0001**
	Maturation category	0.159	2	34.15	**<0.0001**
	Interaction	0.004	4	0.4435	0.7767
Trial 2 K	Photoperiod treatment	0.033	2	13.39	**<0.0001**
	Maturation category	0.071	2	29.15	**<0.0001**
	Interaction	0.011	4	2.303	0.0682

Significant (α = 0.05) *P* values are in bold

**Figure 4:**
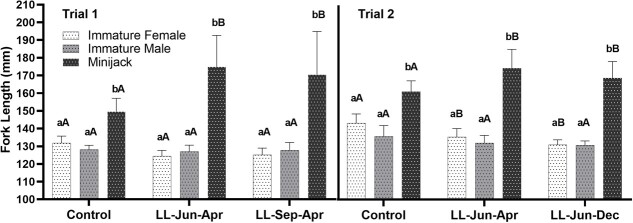
Comparisons of spring Chinook Salmon (*Oncorhynchus tshawytscha*) FL (mean ± SE) between treatment and maturation category for trial 1 (left panel) and trial 2 (right panel) in juvenile spring Chinook Salmon measured at final samplings. Lowercase letters indicate comparisons between maturation categories within a photoperiod treatment group (bars not sharing a letter differ significantly; one-way ANOVA followed by Tukey’s multiple comparison tests, *P* < 0.05). Uppercase letters indicate comparisons between photoperiod treatment groups within each maturation category (significance indication and statistical analysis same as for lowercase letters).

Results from two-way ANOVA showed that maturation category and the interaction between category and treatment had a strong effect on FL (Table 1). There was not a significant effect of treatment on FL (*P* = 0.1647). Like BW, the FL of MJs was significantly greater than IFs and IMs in all treatments at the end of each trial (Figure 4). In trial 1, FL of MJs in LL-Jun-Apr and LL-Sep-Apr (174.9 and 170.5 mm respectively; one-way ANOVA followed by Tukey’s multiple comparison test, *P* < 0.001) were significantly longer than in the control (149.6 mm). Also, in trial 1 no significant difference in FL was observed between IFs and IMs regardless of treatment (one-way ANOVA followed by Tukey’s multiple comparison test, *P* > 0.05). Like trial 1, MJs in trial 2 light treatments (LL-Jun-Apr = 174.0 mm and LL-Jun-Dec = 168.4 mm) were longer than MJs in the control treatment (160.8 mm, one-way ANOVA followed by Tukey’s multiple comparison test, *P* = 0.0003 and 0.0481, respectively). IFs in the trial 2 control group (143.1 mm) were significantly longer than IFs in LL-Jun-Apr and LL-Jun-Dec treatments (135.3, and 130.8 mm, one-way ANOVA followed by Tukey’s multiple comparison test, *P* = 0.0401 and 0.0007, respectively). Lastly, in trial 2, no significant differences between IFs and IMs were detected when comparing within treatment groups (*P* > 0.05).

**Figure 5:**
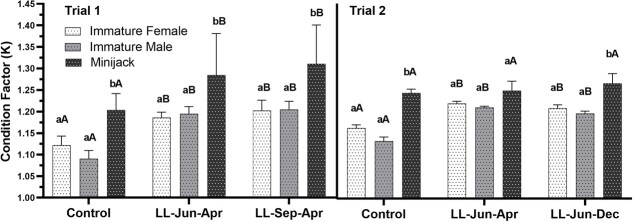
Comparisons of spring Chinook Salmon (*Oncorhynchus tshawytscha*) K (mean ± SE) between treatment and maturation categories for trial 1 (left panel) and trial 2 (right panel) measured at final samplings. Lowercase letters indicate comparisons between maturation categories within a photoperiod treatment group (bars not sharing a letter differ significantly; one-way ANOVA followed by Tukey’s multiple comparison tests, *P* < 0.05). Uppercase letters indicate comparisons between photoperiod treatment groups within each maturation category (significance indication and statistical analysis same as for lowercase letters).

For both trial 1 and 2, two-way ANOVA revealed no significant interaction effect for K. However, maturation category and treatment had a significant effect on final K (Table 2). The data were analysed and presented as for BW and FL for consistency. In trial 1, K for MJs in LL-Jun-Apr (1.28) and LL-Sep-Apr (1.31) was significantly increased compared to the control (1.20; one-way ANOVA followed by Tukey’s multiple comparison test, *P* = 0.0036 and 0.0001, respectively; Figure 5). There was no significant difference in K for MJs in trial 2 between light treatment and control (One-way ANOVA followed by Tukey’s multiple comparison test, LL-Jun-Apr: *P* = 0.9512, LL-Jun-Dec: *P* = 0.4256). In trial 1, IFs and IMs in the continuous light treatments had increased K compared to IFs and IMs in the control treatment (one-way ANOVA followed by Tukey’s multiple comparison test, IFs: LL-Jun-Apr, *P* = 0.0271, LL-Sep-Apr, *P* = 0.0039; IMs: LL-Jun-Apr, *P* = 0.0002, LL-Sep-Apr, *P* < 0.0001). Likewise in trial 2, IFs and IMs in the continuous light treatments had increased K compared to IFs and IMs in the control treatment (one-way ANOVA followed by Tukey’s multiple comparison test, IFs: LL-Jun-Apr *P* = 0.0053, LL-Jun-Dec *P* = 0.0299; IMs: LL-Jun-Apr *P* = 0.0001, LL-Jun-Dec *P* = 0.0015). K for MJs was significantly higher than IMs and IFs within all treatments except in trial 2 LL-Jun-Apr.

There were no differences in BW, FL and K between treatment groups at the intermediate sampling for trial 1 in September (combined BW = 10.2 g, FL = 93.9 mm, K = 1.2). However, by the December sampling, the control group had a higher BW and FL (BW = 15.7 g, FL = 110.7 mm) relative to the light treatment groups (LL-Jun-Apr BW = 13.0 g, FL = 104.7 mm; LL-Sept BW = 13.3 g, FL = 107.0 mm, one-way ANOVA followed by Tukey’s multiple comparison test *P* < 0.0001). No differences in size parameters were observed at the intermediate sampling in December for trial 2 (combined BW = 18.9 g, FL = 115.0 mm, K = 1.2).

## Discussion

### Continuous light treatments reduce minijack proportions

This study shows that partial-year continuous light treatment strongly reduces the proportion of age 1+ male spring Chinook Salmon with elevated 11-KT in the spring, which indicates a corresponding reduction in fish precociously maturing as 2-year-old MJs the following fall (Medeiros *et al.*, 2018). All continuous light regimes resulted in significant decreases in the proportion of MJs compared to fish on a simulated natural photoperiod. To our knowledge, only two previous studies have been conducted that found photoperiodic effects on precocious maturation in Chinook Salmon. However, these were conducted under different circumstances and with different goals from the present study. In California winter Chinook Salmon, an unusual population in which juveniles emerge from late summer to fall, earlier shifted photoperiods begun in the fall at ~3 months post-fertilization increased microjack maturation 9 months later (Beckman *et al.*, 2007). In a stock of Chinook Salmon introduced into New Zealand and raised under production aquaculture conditions, a continuous light photoperiod beginning in the fall at age 1 and continuing for nearly a full year reduced precocious maturation at age 2 in both males and females (Unwin *et al.*, 2005). However, the continuous light photoperiod used in the New Zealand study would be impossible in a conservation hatchery setting such as CESRF, where fish are released at age 1+ in the spring. Thus, while these previous studies demonstrate that photoperiod manipulation can influence precocious maturation in Chinook Salmon, they provide little guidance on how to apply photoperiod manipulation with the goal of reducing MJ maturation in conservation hatcheries for spring Chinook Salmon.

### Potential effects of continuous light treatments on smoltification

Like its role in reproduction, photoperiod is the key environmental cue regulating smoltification, the suite of behavioural, morphological, and physiological changes that prepare juvenile salmonids for downstream migration and seawater entry (McCormick, 2013). Smoltification includes the development of hypoosmoregulatory ability, which is associated with increases in gill Na^+^/K^+^-ATPase enzyme activity (NKA) and a decrease in condition factor (K), along with other changes (McCormick, 2013). Peak levels of NKA occur during May in Yakima River spring Chinook Salmon, whereas the smoltification-related decrease in K begins in early April (Beckman *et al.*, 2000; Larsen *et al.*, 2010). Juveniles released from CESRF need to smolt properly to return as anadromous adults. Studies in Atlantic Salmon suggest that juveniles reared under continuous light can smolt properly, as long as the continuous light is followed by a “winter” of at least 6 weeks of short photoperiod followed by a switch to long photoperiod (Duston and Saunders, 1990; Duncan and Bromage, 1998; Strand *et al.*, 2018). Thus, the LL-Jun-Apr and LL-Sep-Apr photoperiods used in the present study might be expected to disrupt smoltification. However, the LL-Jun-Dec photoperiod allowed 13 weeks of naturally increasing short photoperiod (<12 hours light per day) followed by 8 weeks of naturally increasing long photoperiod (>12 hours light per day) before the expected NKA peak in Yakima River spring Chinook Salmon. Thus, there is reason to think that smoltification may be properly entrained in this group. Juvenile Chinook Salmon raised under a naturally increasing photoperiod showed stronger and more coordinated changes in indices associated with smoltification versus fish raised under a continuous light or a constant photoperiod (Hoffnagle and Fivizzani, 1998). Although condition factor was increased versus controls in all of the continuous light treatments, including the LL-Jun-Dec group, photoperiod manipulation may cause different aspects of smoltification to become desynchronized, without impeding the ability of fish to osmoregulate and grow in saltwater (Duston and Saunders, 1990; Duncan *et al.*, 1998). In addition, the late March to early April final sampling point in the present study was before K decreases in smolting Yakima River spring Chinook salmon and thus would not capture any decrease associated with smolting (Beckman *et al.*, 2000; Larsen *et al.*, 2010). The present study was designed to determine whether photoperiod treatment can reduce precocious male maturation at CESRF, not as an assessment of the effects on smoltification. Investigations evaluating the effects of continuous light regimes on smoltification are necessary before any conclusions can be drawn regarding the usefulness of photoperiod manipulation as a management tool.

### Method to reduce minijack proportions while limiting genetic divergence

Integrated conservation hatcheries are managed to recover and enhance populations of salmonids while limiting genetic divergence from natural origin fish. To meet production goals, the hatchery programme at CESRF, which provided the juvenile Chinook Salmon for this study, uses gametes harvested from natural origin adults returning to the Yakima River, WA each year. The juveniles produced at CESRF are then released into the Yakima River and returning adults of both hatchery and natural origin are allowed to spawn in the river. This type of program contrasts with segregated hatchery broodstock management programs, which utilize hatchery origin adults as broodstock across multiple generations. In order to compare the effects of integrated and segregated broodstock management, as well as any effects of a single generation of hatchery rearing, CESRF produces two lines of juvenile Chinook Salmon: (i) an integrated production line in which the broodstock are comprised of natural-origin returning adults descended from parents that spawned in the wild, and (ii) a segregated hatchery control line for which broodstock are hatchery-origin fish (of increasing hatchery lineage) and for which returning adults are not allowed to spawn in the river (Fast *et al.*, 2015; Waters *et al.*, 2015). To assess any effects of a single generation of hatchery rearing, some of the returning adults from the integrated line (designated as supplementation hatchery or SH line fish) may be used to propagate juveniles to study potential effects of a single generation of hatchery rearing on reproductive parameters and juvenile growth, after which the juveniles are culled (Knudsen *et al.*, 2006, 2008). As these juveniles are never released, they are available for research and are the fish used in the current study.

At a broader scale, a study that investigated the proportion of MJs produced among integrated and segregated spring Chinook Salmon hatcheries across the Columbia River basin showed that integrated hatchery lines produced higher percentages of MJs (Harstad *et al.*, 2014). In agreement with this study, the MJ maturation proportion is higher in the CESRF integrated and supplementation hatchery lines than in the segregated line (Larsen *et al.*, 2019). Therefore, we believe that the supplementation hatchery line used in our study is representative of the integrated production line, at least insofar as response to photoperiod is concerned. The difference in MJ proportion between integrated and segregated lines may be due to differences in hatchery broodstock management, especially given that age at maturity is under relatively strong genetic control in Chinook Salmon (Hankin *et al.*, 1993). Segregated hatcheries select against precocious males across multiple generations, which is proposed to lead to reduced expression of this phenotype (Harstad *et al.*, 2014; Larsen *et al.*, 2019). In contrast, the natural origin fish used as broodstock in integrated programs are part of the naturally spawning population, which includes some precocious male ancestry. This is proposed to result in higher proportions of MJs when progeny from these parents are raised in the hatchery environment (Harstad *et al.*, 2014; Larsen *et al.*, 2019). Thus, the results from this study present a potentially effective method for reducing the proportion of MJs produced by integrated hatchery programmes, without compromising broodstock management designed to minimize genetic divergence between the hatchery reared and natural population.

### Effect of light treatment on size

Some past investigations on smolt to adult returns in Chinook Salmon have shown that larger smolts return at higher rates than their smaller counterparts (Martin and Wertheimer, 1989; Beckman *et al.*, 1999; Beckman *et al.*, 2017), whereas others have not (Feldhaus *et al.*, 2016). For this and other reasons, hatcheries often aim to release large smolts, making it desirable that the methods used to reduce precocious maturation do not reduce the size of released immature fish. Previous studies aimed at reducing MJ proportions in Yakima River spring Chinook Salmon used reduced ration during summer and fall months, which resulted in reduced size of immature fish relative to fully fed controls (Larsen *et al.*, 2006, 2013). The continuous light treatments in this study had little consistent effect on weight or lengths of immature males or females in either trial at the end of the study in April. Therefore, it seems unlikely that the photoperiod treatment would affect the ability of hatchery managers to use previously established protocols to obtain their target release size.

### Differences in %MJ between trials

A large difference in the percentage of precociously maturing fish in the control treatments occurred between the two trials in this study. This result is best explained by a strong effect from the different family genetic backgrounds of the individual broodstock used to produce the fish used in these experiments. Galbreath *et al.* (2022) similarly report a wide range in MJ proportions between family crosses across three brood years of the SH line. Estimated MJ rates from generalized linear models ranged from 20% to 80%, and this high inter-family variation was found to be largely driven by random effects of the individual dams and sires (Galbreath *et al.*, 2022). This suggests heritable factors likely influence precocious maturation rates, and as such, year to year variation in the proportion of MJs is expected given that a different subset of returning adults are captured for spawning at CESRF each year. Furthermore, the variation in the proportion of precociously maturing Chinook Salmon males at integrated hatcheries (including CESRF) has been previously reported to range from 8% to 71% (Larsen *et al.*, 2004; Larsen *et al.*, 2013; Harstad *et al.*, 2014). As the proportions of precociously maturing fish in the control treatments of this study were within this range, it is assumed to be in line with the natural variation between years rather than an effect of the treatment or environmental conditions.

## Conclusion

This study shows that partial-year continuous light treatments significantly reduce precocious male maturation as MJs in spring Chinook Salmon while maintaining the genetic diversity present in the natural origin fish. Additionally, these continuous light treatments generally did not affect immature fish weight or length relative to control treatments, which allows the target size at release to still be under the control of the protocols in place at conservation hatcheries. However, further investigations into the effect of these light treatments on smoltification, sex ratios and returning age class structure must be conducted to fully evaluate the effectiveness of this tool within the salmon hatchery management sphere.

## Funding

This study was supported by funding from the Bonneville Power Administration under project 2009-009-00.

## Data availability

Data generated during the course of this study are available through the corresponding author upon request.

## Supplementary Material

Web_Material_coac085
