# Supplementary material for: Partial-year continuous light treatment reduces precocious maturation in age 1+ hatchery–reared male spring Chinook Salmon (Oncorhynchus tshawytscha)
Source: Conserv Physiol. 2023 Jan 20;11(1):coac085. doi: 10.1093/conphys/coac085 (PMC9868527; doi:10.1093/conphys/coac085)
Supplement: Web_Material_coac085 [file web_material_coac085.docx]

| **Trial 1** | **Total Males** | **Total IM** | **Total MJ** |  |
| --- | --- | --- | --- | --- |
|  |  |  |  | **Total Females** |
| Control | 321 | 229 | 92 | 311 |
| LL-Jun-Apr | 352 | 333 | 19 | 318 |
| LL-Sep-Apr | 321 | 291 | 30 | 318 |
| **Trial 2** |  |  |  |  |
| Control | 338 | 115 | 223 | 331 |
| LL-Jun-Apr | 329 | 295 | 34 | 292 |
| LL-Jun-Dec | 343 | 303 | 40 | 329 |

**Supplementary Table 1.** Total number of immature males (IM), females, and minijacks (MJ) spring Chinook Salmon (*Oncorhynchus tshawytscha)* sampled at the end of each trial

**Supplementary Figures**

Supplementary Figure 1


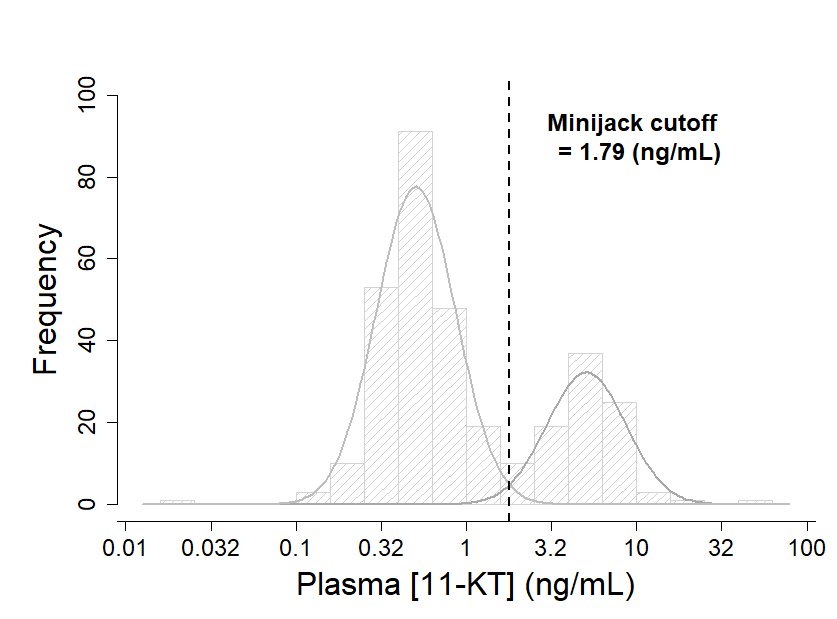


**Supplementary Figure 1.** Significant bimodal 11-KT frequency histograms of O*ncorhynchus tshawytscha* from trial 1 control group. Lower mode (below minijack cutoff value, dotted line) represents 11-KT values from immature males (IM) and the upper mode (above minijack cutoff value) is 11-KT values from minijacks (MJ).

Supplementary Figure 2


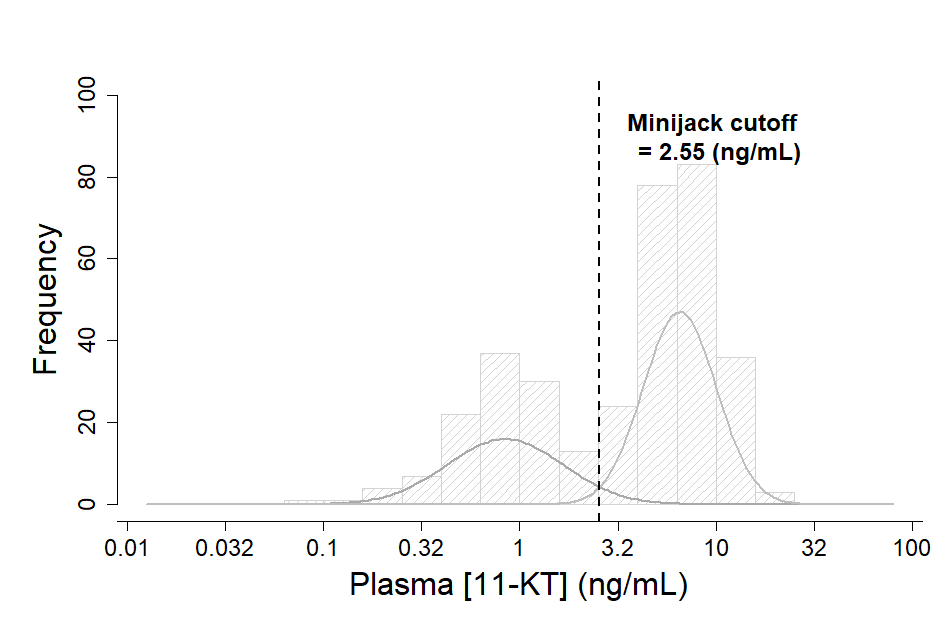

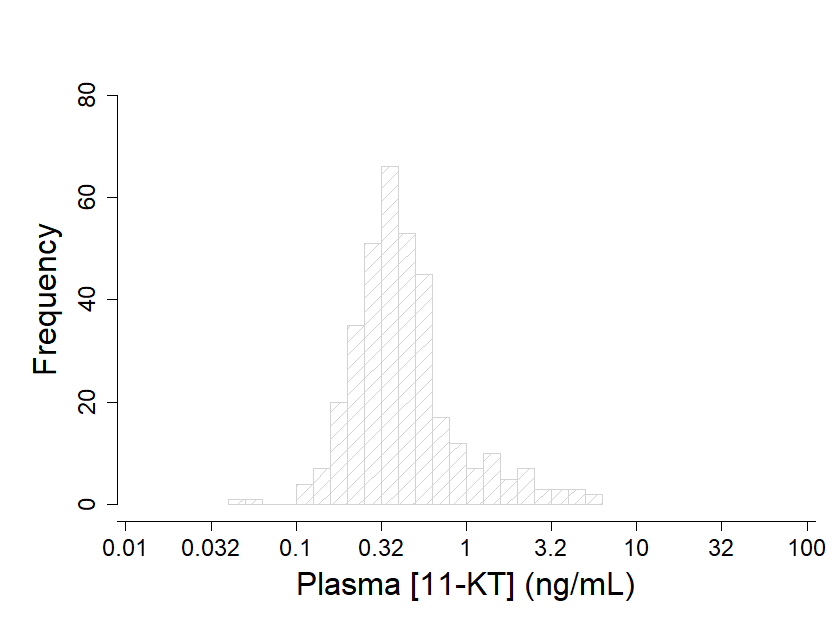


**Supplementary Figure 2.** Significant bimodal 11-KT frequency histograms of O*ncorhynchus tshawytscha* from trial 2 control group. Lower mode (below minijack cutoff value, dotted line) represents 11-KT values from immature males (IM) and the upper mode (above minijack cutoff value) is 11-KT values from minijacks (MJ).


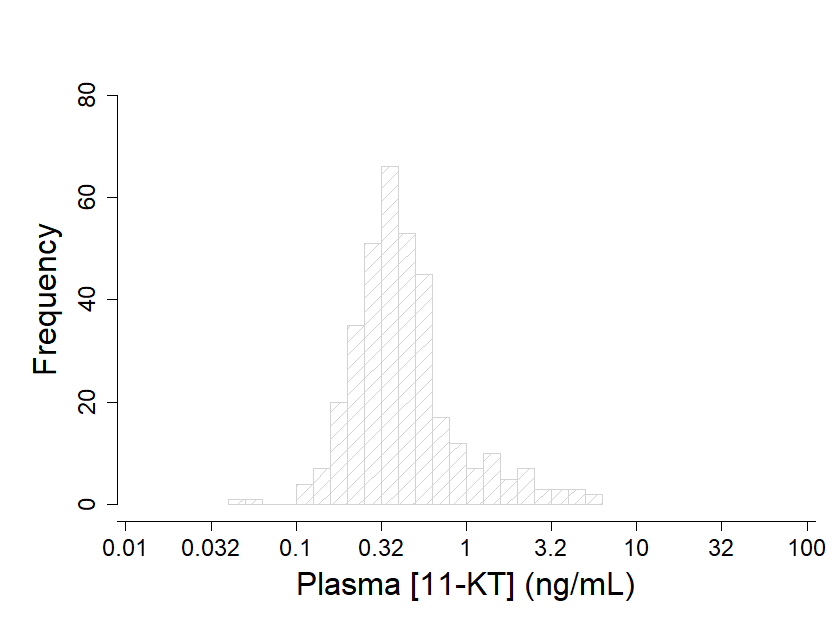
Supplementary Figure 3

**Supplementary Figure 3.** Non-significant bimodal 11-KT frequency histograms of O*ncorhynchus tshawytscha* from trial 1 LL-Jun-Apr treatment.

Supplementary Figure
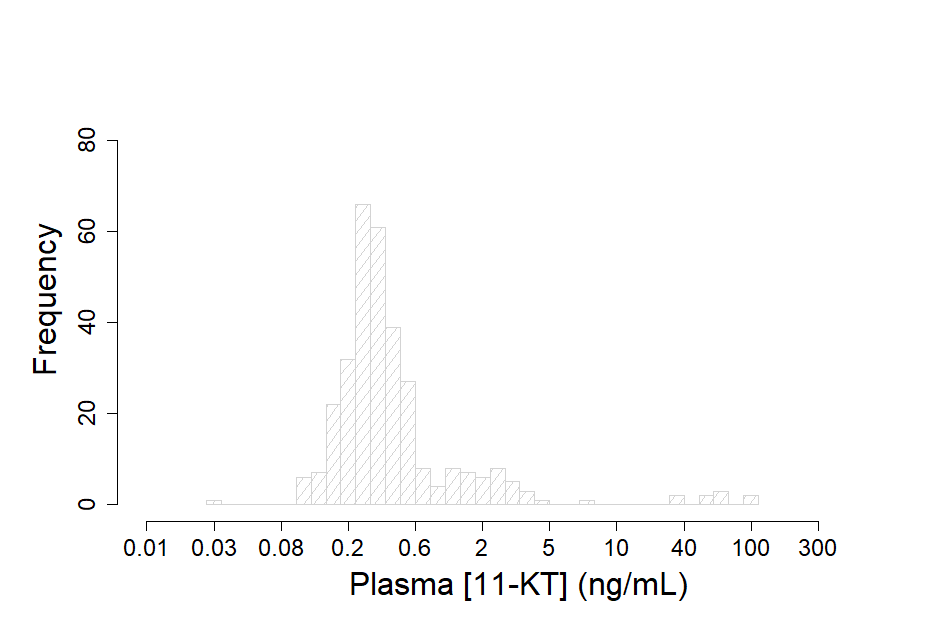
4

**Supplementary Figure 4.** Non-significant bimodal 11-KT frequency histograms of O*ncorhynchus tshawytscha* from trial 1 LL-Sep-Apr treatment.

Supplementary Figure 5


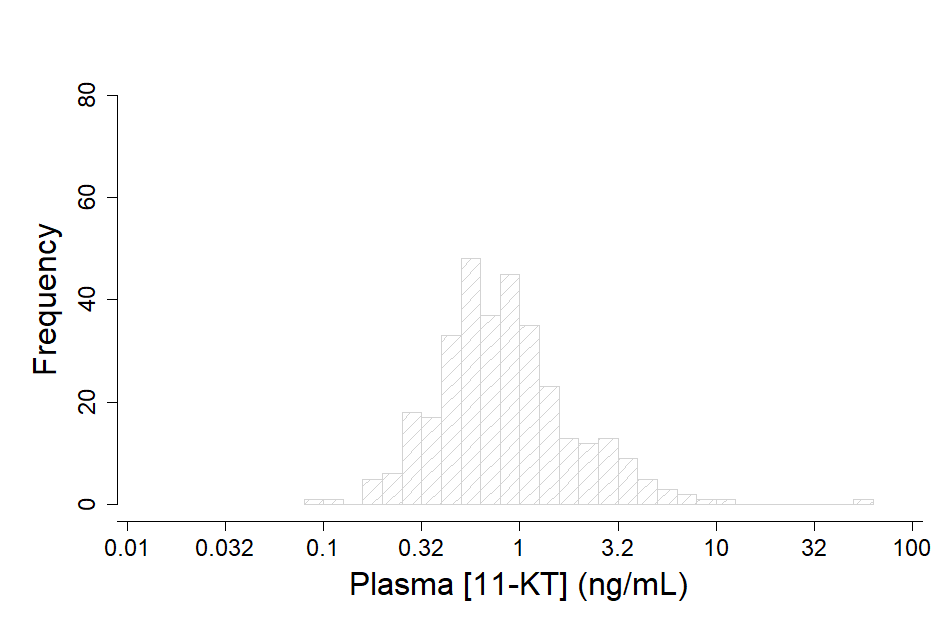


**Supplementary Figure 5.** Non-significant bimodal 11-KT frequency histograms of O*ncorhynchus tshawytscha* from trial 2 LL-Jun-Apr treatment.

Supplementary Figure 6


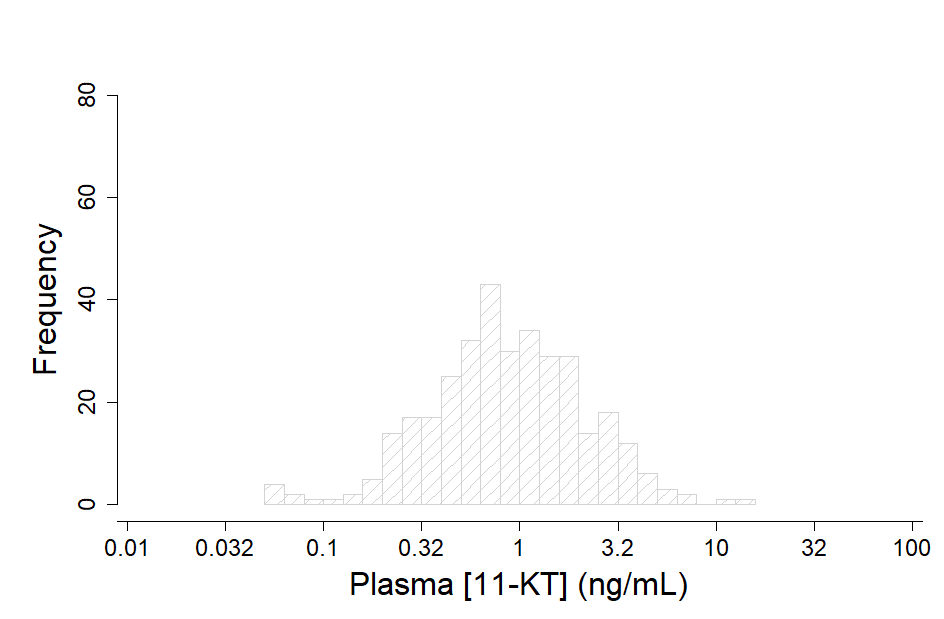


**Supplementary Figure 6.** Non-significant bimodal 11-KT frequency histograms of O*ncorhynchus tshawytscha* from trial 2 LL-Jun-Dec treatment.
